# Interactions between Plasma Levels of 25-Hydroxyvitamin D, Insulin-Like Growth Factor (IGF)-1 and C-Peptide with Risk of Colorectal Cancer

**DOI:** 10.1371/journal.pone.0028520

**Published:** 2011-12-28

**Authors:** Kana Wu, Diane Feskanich, Charles S. Fuchs, Andrew T. Chan, Walter C. Willett, Bruce W. Hollis, Michael N. Pollak, Edward Giovannucci

**Affiliations:** 1 Department of Nutrition, Harvard School of Public Health, Boston, Massachusetts, United States of America; 2 Channing Laboratory, Department of Medicine, Brigham and Women's Hospital, Harvard Medical School, Boston, Massachusetts, United States of America; 3 Gastrointestinal Unit, Massachusetts General Hospital and Harvard Medical School, Boston, Massachusetts, United States of America; 4 Division of Medical Oncology, Dana-Farber Cancer Institute, Boston, Massachusetts, United States of America; 5 Department of Epidemiology, Harvard School of Public Health, Boston, Massachusetts, United States of America; 6 Division of Pediatrics, Medical University of South Carolina, Charleston, South Carolina, United States of America; 7 Department of Medicine and Oncology, Jewish General Hospital and McGill University, Montreal, Quebec, Canada; Sun Yat-sen University Cancer Center, China

## Abstract

**Background:**

Vitamin D status and levels of insulin-like growth factor (IGF)-1 and C-peptide have been implicated in colorectal carcinogenesis. However, in contrast to vitamin D IGF-1 is not an easily modifiable risk factor.

**Methods:**

Combining data from the Health Professionals Follow up Study (HPFS) and the Nurses' Health Study cohort (NHS) additive and multiplicative interactions were examined between plasma 25-hydroxyvitamin D (25(OH)D) and IGF-1, IGFBP-3 as well as C-peptide levels in 499 cases and 992 matched controls. For the various analytes, being high or low was based on being either above (or equal) or below the medians, respectively.

**Results:**

Compared to participants with high 25(OH)D and low IGF-1/IGFBP-3 ratio (reference group), participants with a high IGF-1/IGFBP-3 ratio were at elevated risk of colorectal cancer when 25(OH)D was low (odds ratio (OR): 2.05 (95% CI: 1.43 to 2.92), but not when 25(OH)D was high (OR:1.20 (95% CI: 0.84 to 1.71, p(interaction): additive  = 0.06, multiplicative  = 0.25). Similarly, compared to participants with high 25(OH)D and low molar IGF-1/IGFBP-3 ratio and low C-peptide levels (reference group), participants with a combination of either high IGF-1/IGFBP-3 ratio or high C-peptide were at elevated risk for colorectal cancer when 25(OH)D was low (OR = 1.90, 95% CI: 1.22 to 2.94) but not when 25(OH)D was high (OR = 1.15, 95% CI: 0.74 to 1.77, p(interaction): additive = 0.004; multiplicative  = 0.04).

**Conclusion:**

The results from this study suggest that improving vitamin D status may help lower risk of colorectal cancer associated with higher IGF-1/IGFBP-3 ratio or C-peptide levels.

## Introduction

Both vitamin D status and levels of insulin-like growth factor (IGF)-1 has been implicated in colorectal carcinogenesis [1]–[3]. Epidemiological studies have provided consistent support for an elevated risk of colorectal cancer with lower plasma 25-hydroxyvitamin D (25(OH)D) levels [4]–[10]. Several but not all prospective studies have found evidence for a positive association between higher levels of IGF-1 or the molar ratio of IGF-1 and its primary binding protein, IGF-binding protein 3 [11] (IGF-1/IGFBP-3 ratio) and colorectal and/or colon cancers [12]–[19]. High levels of insulin (or C-peptide, a marker for insulin production [20]) may also increase risk of colorectal cancer [13], [16], [21]–[23]. The IGF axis and the insulin axis are biologically interconnected; for example, insulin may decrease IGF-BP1 levels [24], [25]. Insulin level is strongly modifiable by lifestyle and diet [26], but IGF-1 is not an easily modifiable risk factor because for total IGF-1 levels modifiable dietary or lifestyle factors identified to date have been associated with differences of a relatively small magnitude [27]–[38].

Laboratory studies have suggested that 1, 25-dihydroxyvitamin D3, the biologically active form of vitamin D, might play a role in the regulation of several IGF binding proteins including the major binding protein IGFBP-3, indicating that some of the cancer promoting effects of IGF-1 may be modified by vitamin D [39]–[41]. In a study by Ma et al. [42] positive associations between IGF-1/IGFBP-3 ratio, suggested to be a better marker for bioavailable IGF-1 [43], and colorectal cancers were most pronounced among participants who never or rarely drank low-fat milk, a primary food source for vitamin D.

In previously published studies using data from the Health Professionals Follow-up Study (HPFS) and the Nurses' Health Study (NHS) cohorts, higher plasma 25(OH)D levels were significantly associated with decreased risk and higher IGF-1 levels, the molar IGF-1/IGFBP-3 ratio and C-peptide levels were associated with increased risk of colorectal and/or colon cancer [6], [8], [21]. In this report, we examined the joint and interactive effects of 25(OH)D, components of the IGF axis (IGF-1, IGFBP-3) and C-peptide, combining data from the HPFS and NHS blood cohorts.

## Materials and Methods

### Study Population

The HPFS cohort was started in 1986 when 51, 129 male U.S. health professionals aged between 40–75 years were mailed a questionnaire inquiring about their medical history and lifestyle factors as well as a 131-item food frequency questionnaire (FFQ). Since then, follow-up questionnaires have been mailed every two years and FFQs have been mailed every 4 years. The NHS cohort was started in 1976 and included 121,700 female nurses residing in the U.S. who had responded to a mailed questionnaire on lifestyle and medical history. In 1980, 1984, 1986 and every four years thereafter participants were also asked to complete a FFQ. More details regarding the main HPFS and NHS cohorts have been published elsewhere (HPFS: [44], [45] , NHS: [46]). Between 1993–1995 the HPFS blood cohort was established when 18,225 participants in this cohort provided blood samples and the NHS blood cohort was established between 1989 and 1990 when 32,826 NHS participants provided blood samples. For more details regarding blood collection, handling and storage of the blood samples please refer to (HPFS: [47], NHS: [48]). For this study we also included laboratory measurements on 134 additional cases from the NHS diagnosed between June 2000 and October 2008 which had not been included in our previous publications on associations between plasma 25(OH)D levels and biomarkers of the IGF and insulin axis and colorectal cancer [6], [8], [21]. Cases were identified by study investigators who reviewed medical and pathology records. Each case was matched to 2 controls by age (within 3 years of birth), year and month of blood donation (95% of cases and controls were matched within one month of blood donation) as well as fasting status (<8 vs. ≥8 hours since last meal, NHS only). Controls were required to be alive and free of any cancer diagnosis (except for non-melanoma skin cancer) at the time of diagnosis of the case. A total of 499 cases (174 incident colorectal cancer cases from the HPFS blood cohort and 325 cases from the NHS blood cohort who were diagnosed after the date of blood draw and up to January 2002 (HPFS) or October 2008 (NHS)) and 992 matched controls were included in our final analysis.

This study was approved by the Committee on the Use of Human Subjects in Research at the Brigham and Women's Hospital as well as the Human Subjects Committee of the Harvard School of Public Health. Return of the questionnaires was considered to imply informed consent and we also obtained written consent from each participant to obtain and review medical records.

### Laboratory Analysis

Plasma 25(OH)D levels were assessed at the laboratory of Dr. Bruce Hollis at the University of South Carolina) by the radioimmunoassay method as described elsewhere [49]. IGF-1, IGFBP-3 and C-peptide levels were determined at the laboratory of Dr. Michael Pollak at the Lady Davis Research Institute of the Jewish General Hospital and McGill University, using ELISA with reagents from the Diagnostic Systems Laboratory (Webster, TX). Each case-control triplet was analyzed in the same batch and the laboratory personnel were blinded with regard to case control status. For quality control purposes plasma from pooled blood samples arranged in triplets were also inserted randomly among the case-control samples. Using those quality control samples all mean intra-pair coefficients of variation for plasma 25(OH)D, C-peptide, IGF-I, and IGFBP-3 were ≤15%. A molar IGF-I/IGFBP-3 ratio (IGF-1: 1 ng/mL = 0.13 nmol; IGFBP-3: 1 ng/mL  = 0.036 nmol) was also calculated in order to better estimate bioavailable IGF-I [43].

### Questionnaire and dietary information

We calculated nutrient intake as the mean from the 1986, 1990 and 1994 FFQs in HPFS and the 1980, 1984, 1986 and 1990 FFQs in NHS [50] (except for calcium and retinol intake which was calculated using information from the 1990 FFQ in NHS and 1994 FFQ in HPFS and if missing using available information from the most recent FFQ prior to blood donation). For body mass index (BMI in kg/m^2^) and physical activity we used information obtained from the questionnaires closest to blood donation, i.e. 1994 in HPFS and 1990 in NHS and if not available from the most recent questionnaire prior to blood donation. All other lifestyle variables were updated up to the time of blood donation, or if not available were carried forward from previous follow-up questionnaires. The FFQs as well as the anthropometric measures and physical activity have been validated in previous studies [51]–[56]. History of diabetes was assessed using information on self-reported diabetes from the biennial follow-up questionnaires in NHS and HPFS. Family history of colorectal cancer was obtained from the biennial follow-up questionnaires using information from the 1986, 1990 and 1992 questionnaires (HPFS) and the 1982 and 1988 questionnaires (NHS).

### Statistical analysis

To increase statistical power to examine our study question and because main effects were similar in both cohorts, data from both blood cohorts were combined. To test whether differences in mean levels of the plasma analytes between cases and controls were statistically significant the Wilcoxon signed rank test was used. To assess the association between medians of each plasma analyte (based on the median within sex and laboratory batch among controls) and colorectal or colon cancer, we employed a conditional logistic regression model. Associations between C-peptide and colorectal and colon cancers as well as interactions between C-peptide and plasma 25(OH)D with regard to colorectal and colon cancers were examined after exclusion of participants with a self-reported history of diabetes mellitus prior to blood donation because in these participants C-peptide levels may not be a good marker for long-term insulin exposure [20]. The following variables were included in the final multivariable models: BMI (kg/m^2^, continuous), pack-years of smoking (continuous), physical activity (METs-hr/week, continuous), intake of alcohol (grams/day), methionine (grams/day), folate (ug/day), retinol (IU/day), red and processed meat (servings/day), calcium (mg/day) (all intake covariates as continuous variables), family history of colorectal cancer (yes vs. no), sex, fasting status (0–2, 3–4, 5–8, ≥9 hours since last meal), aspirin use (<2 tablets/week, past use, ≥2 tablets/week). In addition, all models for IGF-1 were adjusted for IGFBP-3 (in tertiles) and vice versa. All p-values were two sided and a p-value of <0.05 was considered statistically significant.

### Assessment of additive interaction

The methodology used to examine additive interaction has been reported in more detail in a previous publication from the NHS cohort [57]. We calculated two measures of additive interaction previously defined by Rothman [58]: the RERI, i.e. the relative excess risk due to (additive) interaction ((RR11-RR10-RR01) + 1)  = 0; if no interaction; RR11 = relative risk among those exposed to both risk factor #1 and risk factor #2, RR10 = relative risk among those exposed to risk factor #1 but not risk factor #2, RR01 = relative risk among those exposed to risk factor #2 but not risk factor #1) and based on RERI AP was calculated, i.e. the proportion of disease among those with both exposures that is attributable to their interaction (RERI / RR11  = 0; if no interaction).We used SAS programs published by Lundberg and Andersson [59], [60] to calculate these measures of additive interaction. Confidence intervals for AP were calculated based on methods described in detail by Hosmer and Lemeshow [61]. A p-value of <0.05 for AP was considered statistically significant for additive interaction.

### Assessment of multiplicative interaction

Multiplicative interactions between IGF-1, IGFBP-3, C-Peptide and plasma 25(OH)D with regard to colorectal and colon cancers were examined by creating binary variables based on the median assignment of IGF-1, IGFBP-3 or C-Peptide levels and median assignment of plasma 25(OH)D levels. Test for multiplicative interaction was evaluated by adding cross term products of the pertinent plasma analyte (as binary variables) to the models and then using the Wald test to calculate the p-value. A p-value of <0.05 was considered statistically significant for multiplicative interaction.

## Results

Cases had significantly higher mean plasma IGF-1 levels and molar IGF-1/IGFBP-3 ratio as well as lower plasma 25(OH)D levels compared to controls (Table 1). Cases and controls did not differ considerably with regard to most baseline characteristics, except that cases were more likely to report a family history of colorectal cancer, were less likely to be aspirin users and had higher intakes of unprocessed red and processed meats combined and lower intake of folate, retinol and calcium. Consistent with results reported in our previous publications on colorectal or colon cancer from our cohorts [6], [8], [21], in multivariable analyses higher IGF-1 and C-peptide levels and IGF-1/IGFBP-3 molar ratio were significantly associated with higher risk of colorectal and colon cancer and higher plasma 25(OH)D levels were significantly associated with lower risk of colorectal and colon cancer (Table 2).

**Table 1:** Mean plasma analyte levels and baseline characteristics for cases and controls Health Professionals Follow-up Study (HPFS), 1994–2002 and Nurses' Health Study (NHS), 1990–2008 (combined).

Characteristics	Cases(N = 499)	Controls (N = 992)	p-value ‡‡‡‡
Plasma 25(OH)D (ng/mL)	26.2 (10.3)	27.6 (9.8)	<0.001
Plasma IGF-1 (ng/mL)	185 (69.7)	175 (66.3)	0.02
Plasma IGF-BP3 (ng/mL)	4352 (1025)	4291 (1013)	0.22
Molar ratio IGF-1/IGF-BP3	0.15 (0.05)	0.15 (0.05)	0.03
Plasma C-Peptide (ng/mL) (excluding participants with history of diabetes)	2.4 (1.6)	2.3 (1.9)	0.52
Sex ‡‡‡‡‡			
Male	174 (34.9)	346 (34.9)	
Female	325 (65.1)	646 (65.1)	1.00
Age at blood donation, years ‡‡‡‡‡	61.7 (8)	61.6 (7.9)	0.95
Hours since last meal ‡‡‡‡‡			
<8 hours	129 (25.9)	248 (25.0)	
≥ 8 hours	335 (67.1)	671 (67.6)	
Missing	35 (7.1)	73 (7.4)	0.92
Season of blood donation‡‡, ‡‡‡‡‡			
Winter	179 (35.9)	361 (36.4)	
Summer	111 (22.2)	213 (21.5)	
Spring/Fall	209 (41.9)	418 (42.1)	0.94
Body mass index (BMI), kg/m^2^ $	26.0 (4.5)	25.5 (4.1)	0.08
Physical activity, MET- hour/wk$	22.6 (26.1)	23.3 (26.9)	0.93
Aspirin use $			
<2 tablets/week	206 (41.3)	325 (32.8)	
≥2 tablets/week	139 (27.9)	318 (32.1)	
Past user	154 (30.9)	349 (35.2)	0.005
Pack-years of smoking ‡	26.2 (22)	24 (19.6)	0.22
Family history of colorectal cancer			
No	410 (82.2)	865 (87.2)	
Yes	89 (17.8)	127 (12.8)	0.009
Alcohol, g/d †	8.7 (11.7)	8.4 (11.6)	0.77
Methionine g/d †	1.90 (0.35)	1.90 (0.34)	
Total folate intake, µg/d †	424 (187)	456 (212)	0.09
Total vitamin D intake , IU/d ‡‡‡	375 (265)	400 (281)	0.14
Vitamin D from supplements only , IU/d ‡‡‡	145 (229)	159 (229)	0.23
Total calcium intake, mg/d ‡‡‡	908 (340)	953 (357)	0.008
Total retinol intake, IU/d ‡‡‡	3886 (4466)	4241 (4462)	0.02
Total fiber intake, g/d †	18.6 (5.6)	19.2 (6.0)	0.12
Processed meat intake, serv/d †	0.32 (0.35)	0.29 (0.30)	0.11
Unprocessed red meat intake, serv/d †	0.59 (0.33)	0.57 (0.32)	0.18
Unprocessed red and processed meat, serv/d †	0.92 (0.57)	0.86 (0.52)	0.07
Total milk intake, serv/d ‡‡‡	0.98 (1.05)	1.0 (0.99)	0.34

*Numbers in parenthesis indicate standard deviation for means and percentages for frequencies.

$If not noted otherwise (see below) information obtained from 1994 questionnaires (HPFS) or 1990 questionnaire (NHS),

if missing carried forward from most recent questionnaire prior to 1994 (HPFS) or prior to 1990 (NHS).

† For dietary intake: cumulative average intake 1986-1994 (HPFS) and 1980-1990 (NHS), up to 9 participants have missing information.

‡ Never smokers (N = 633) and participants with missing values (N = 16) were excluded when calculating means.

‡‡ Season defined as: winter: November, December, January, February, March, summer: June, July, August, September, spring/fall : all other months.

‡‡‡ Intake calculated from FFQ in 1994 (HPFS) and FFQ in 1990 (NHS), if missing carried forward from most recent questionnaire prior to 1994 (HPFS) or prior to 1990 (NHS) up to 9 participants have missing information.

‡‡‡‡ P-values calculated using Wilcoxon signed rank test for plasma biomarkers, Wilcoxon rank sum test for other continuous variables, and χ2 tests for categorical variables.

‡‡‡‡‡ Matching factor

**Table 2:** Multivariable adjusted odds ratios (OR) of colorectal and colon cancer by median of plasma analyte level, HPFS and NHS combined.

	*Colorectal Cancer*	**	Colon Cancer	
Plasma Marker	Low*	High*	Low	High
**IGF-1**				
Cases/controls	220/497	279/495	156/360	210/367
OR (95% CI)$	1.00	1.37 (1.05 to 1.78)	1.00	1.52 (1.11 to 2.07)
**IGFBP3**				
Cases/controls	230/496	269/496	171/362	195/365
OR (95% CI)	1.00	0.96 (0.74 to 1.26)	1.00	0.91 (0.68 to 1.25)
**Molar IGF-1/IGFBP3 Ratio**				
Cases/controls	219/497	280/495	159/353	207/374
OR (95% CI)	1.00	1.36 (1.08 to 1.73)	1.00	1.35 (1.02 to 1.79)
**C-Peptide** †				
Cases/controls	197/455	277/460	140/337	211/339
OR (95% CI)	1.00	1.37 (1.05 to 1.78)	1.00	1.58 (1.16 to 2.16)
**Vitamin D**				
Cases/controls	*302/498*	*197/494*	224/354	142/373
OR (95% CI)	*1.00*	*0.67 (0.53 to 0.86)*	1.00	0.62(0.47 to 0.83)

*Low: below median level; high: equal or above median level.

Median vitamin D levels (controls): HPFS: 29.6 ng/mL; NHS: laboratory batch 1: 23.8 ng/mL; laboratory batch 2: 29.7 ng/mL, laboratory batch 3: 25.5 ng/mL.

$Multivariable adjusted for matching factors (age, year and month of blood donation), body mass index (kg/m2, continuous), packyears of smoking (continuous), physical activity (MET-hr week, continuous), intake of alcohol, methionine, folate, retinol, red and processed meat, calcium (all intake covariates as continuous variables), family historyof colorectal cancer (yes vs. no), sex, fasting status (0–2, 3–4, 5–8, ≥9 hours since last meal), aspirin use (<2 tablets/week, past, ≥2 tablets/week); models for IGF-1 were adjusted for IGF-BP3 (in tertiles) and vice versa.

† Models for C-Peptide only included participants without self-reported diabetes mellitus.

Compared to participants with high 25(OH)D and low IGF-1/IGFBP-3 ratio (reference group), participants with a high IGF-1/IGFBP-3 ratio were at elevated risk of colorectal and colon cancers when 25(OH)D was low (colorectal: odds ratio (OR): 2.05 (95% CI: 1.43 to 2.92), colon: OR: 2.26 (95% CI: 1.48 to 3.47) but not when 25(OH)D was high (colorectal: OR: 1.20 (95% CI: 0.84 to 1.71, p(interaction): additive  = 0.06, multiplicative  = 0.25, colon: OR: 1.27 (95% CI: 0.84 to 1.94), p(interaction): additive  = 0.20, multiplicative  = 0.55), Table 3). A similar pattern was observed for IGF-1. Similarly, compared to participants with high 25(OH)D and low C-peptide levels (reference group), risk of colorectal and colon cancers were also elevated when 25(OH)D was low (colorectal: OR: 1.90 (95% CI: 1.32 to 2.75), colon: OR: 2.31 (95% CI: 1.49 to 3.58), but not when 25(OH)D was high (colorectal: OR: 1.18 (95% CI: 0.82 to 1.70), p(interaction); additive  = 0.10; multiplicative  = 0.28, colon: OR: 1.25 (95% CI: 0.81 to 1.91), p(interaction); additive  = 0.01; multiplicative  = 0.11).

**Table 3:** Multivariable adjusted ORs and 95% confidence intervals of colorectal and colon cancer by median levels of IGF-1, C-peptide, IGF-1/IGFBP3 ratio and vitamin D, HPFS and NHS combined.

OR (95% CI) (Cases/Controls)	Colorectal Cancer		Colon Cancer	
	**IGF-1**		**IGF-1**	
**Vitamin D**	**Low**	**High**	**Low**	**High**
**High**	1.00	1.30	1.00	1.58
	(85/238)	(0.89 to 1.89)	(58/183)	(1.02 to 2.47)
		(112/256)		(84/190)
**Low**	1.42 (0.99 to 2.02)	2.04	1.69	2.53
	(135/256)	(1.41 to 2.96)	(1.09 to 2.62)	(1.62 to 3.95)
		(167/239)	(98/177)	(126/177)
	**Additive IA** ‡‡	**Multiplicative IA**	**Additive IA**	**Multiplicative IA**
p- interaction	0.29	0.65	0.57	0.84
AP‡‡	0.16		0.10	
	(-0.14 to 0.46)		(-0.25 to 0.45)	**Molar IGF-1/IGFBP3 ratio**		**Molar IGF-1 /IGFBP3 ratio**	
**Vitamin D**	**Low**	**High**	**Low**	**High**
**High**	1.00	1.20	1.00	1.27
	(86/228)	(0.84 to 1.71)	(60/170)	(0.84 to 1.94)
		(111/266)		(82/203)
**Low**	1.31	2.05	1.50	2.26
	(0.92 to 1.87)	(1.43 to 2.92)	(0.98 to 2.30)	(1.48 to 3.47)
	(133/269)	(169/229)	(99/183)	(125/171)
	**Additive IA**	**Multiplicative IA**	**Additive IA**	**Multiplicative IA**
p- interaction	0.06	0.25	0.20	0.55
AP	0.26		0.22	
	(-0.01 to 0.54)		(-0.12 to 0.55)	
	**C- peptide**		**C- peptide**	
**Vitamin D**	**Low**	**High**	**Low**	**High**
**High**	1.00	1.18	1.00	1.25
		(0.82 to 1.70)		(0.81 to 1.91)
	(91/240)	(97/213)	(65/179)	(71/163)
**Low**	1.25	1.90	1.18	2.31
	(0.87 to 1.80)	(1.32 to 2.75)	(0.76 to 1.83)	(1.49 to 3.58)
	(106/215)	(180/247)	(75/158)	(140/176)
	**Additive IA**	**Multiplicative IA**	**Additive IA**	**Multiplicative IA**
p- interaction	0.10	0.28	0.01	0.11
AP	0.25		0.39	
	(-0.05 to 0.55)		(0.09 to 0.68)	

*Low: below median level; high: equal or above median level.

$Multivariable adjusted for matching factors (age, year and month of blood donation), body mass index (kg/m2, continuous), pack-years of smoking (continuous), physical activity (MET-hr week, continuous), intake of alcohol, methionine, folate, retinol, red and processed meat, calcium (all intake covariates as continuous variables), family history of colorectal cancer (yes vs. no), sex, fasting status (0–2, 3–4, 5–8, ≥9 hours since last meal), aspirin use (<2 tablets/week, past, ≥2 tablets/week); models for IGF-1 were adjusted for IGF-BP3 (in tertiles) and vice versa

† Models for C-Peptide only included participants without self-reported diabetes mellitus.

‡‡ Abbreviations: IA = interaction, AP  = attributable proportion due to interaction, RERI =  relative excess risk due to interaction, RR11 = relative risk among those exposed to both risk factor #1 and risk factor #2, RR10 = relative risk among those exposed to risk factor #1 but not risk factor #2, RR01 = relative risk among those exposed to risk factor #2 but not risk factor #1

AP =  RERI / RR11 ( = 0 if no interaction) with RERI =  RR11 - RR10 - RR01 + 1 ( = 0 if no interaction)

**Figure 1:**
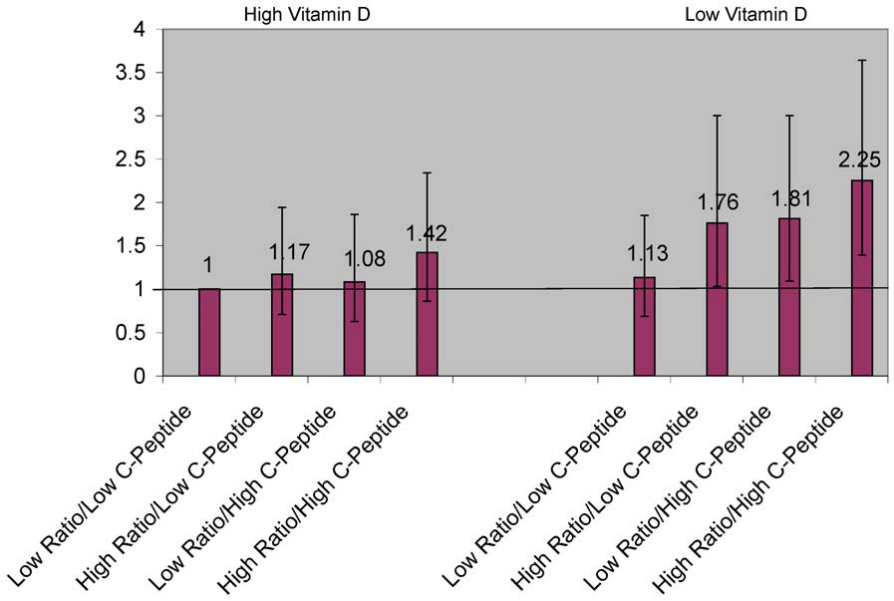
Multivariate odds ratios and 95% confidence intervals of colorectal cancer by different combinations of plasma 25(OH)D, IGF-1/IGFBP3 ratio (ratio) and C-peptide (high = above or equal median; low  =  below median), HPFS and NHS combined.

**Figure 2:**
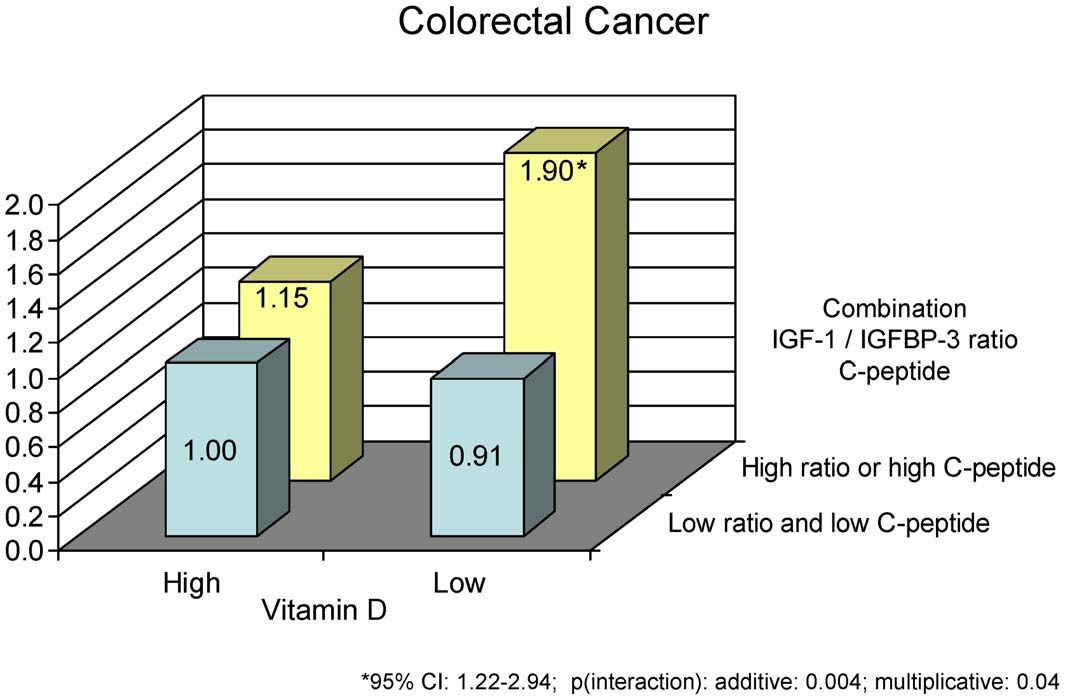
Multivariate adjusted odds ratios and 95% confidence intervals of colorectal cancer by plasma 25(OH)D levels and combinations of C-peptide levels and molar IGF-1/IGFBP3 ratio, HPFS and NHS combined.

Considering previous findings from the NHS blood cohort which suggested that among women with high IGF-1/IGFBP-3 ratio having also high C-peptide levels did not further increase risk of colorectal cancer and vice versa [62], we examined associations between different combinations of molar IGF-1/IGFBP-3 ratio and C-peptide and plasma 25(OH)D and risk of colorectal cancers using our combined HPFS and NHS dataset. As shown in Figure 1, we found that those with high 25(OH)D did not have a significant increase in risk regardless of IGF-1/IGFBP-3 ratio or C-peptide level, whereas among those with low 25(OH)D, the participants with high IGF-1/IGFBP-3 ratio or C-peptide were at elevated risk. Our findings also suggest that among those with low plasma 25(OH)D levels, having either high IGF-1/IGFBP-3 ratio or high C-peptide levels increased risk, but being high in both did not further increase colorectal cancer risk appreciably. Therefore, to increase statistical efficiency, we created another binary variable combining molar IGF-1/IGFBP-3 ratio and C-peptide levels (0 =  molar IGF-1/IGFBP-3 ratio below median and C-peptide below median; 1 =  molar IGF-1/IGFBP-3 ratio above or equal to median or C-peptide above or equal to median) and examined additive and multiplicative interactions between this combined variable and plasma 25(OH)D levels (above or equal median vs. below median) with regard to colorectal cancer risk (Figure 2). Compared to participants with high 25(OH)D and low molar IGF-1/IGFBP-3 ratio and low C-peptide levels (reference group), participants with a combination of either high IGF-1/IGFBP-3 ratio or high C-peptide had an almost 2 fold elevated risk for colorectal and colon cancer (colorectal: OR: 1.90 (95% CI: 1.22 to 2.94, colon: OR: 2.05 (95% CI: 1.23 to 3.41), when 25(OH)D was low, but not when 25(OH)D was high (colorectal: OR: 1.15 (95% CI: 0.74 to 1.77), p(interaction); additive  = 0.004; multiplicative  = 0.04, colon: OR: 1.13 (95% CI: 0.69 to 1.86), p(interaction): additive <0.001; multiplicative  = 0.02).

Results were similar albeit slightly attenuated compared to those presented in Figure 2 after we excluded cases diagnosed up to 2 years after blood donation (high IGF-1/IGFBP-3 ratio or high C-peptide vs. high 25(OH)D and low molar IGF-1/IGFBP-3 ratio and low C-peptide levels (reference group): colorectal: OR: 1.83 (95% CI: 1.15 to 2.91, p(interaction); additive 0.02, multiplicative = 0.09; colon: OR: 1.96 (95% CI: 1.14 to 3.38, p(interaction); additive 0.008, multiplicative = 0.07).

## Discussion

In this combined prospective study using nested case-control data from the HPFS and NHS, we found evidence for statistically significant additive and multiplicative interactions between plasma 25(OH)D and plasma levels of some components of the IGF axis and C-peptide with regard to risk of colorectal and colon cancer. In particular, participants with high 25(OH)D and high C-peptide or high molar IGF-1/IGFBP-3 ratio did not have a significantly increased risk of colorectal cancer whereas those with a combination of low 25(OH)D and high C-peptide or high molar IGF-1/IGFBP-3 ratio had an about 2 fold increased risk of developing colorectal cancer when compared to participants with high 25(OH)D and low C-peptide and low molar IGF-1/IGFBP-3 ratio. Overall, low 25(OH)D levels appeared to be less of a risk factor when both IGF-1/IGFBP-3 ratio and C-peptide levels were low.

Several but not all prospective studies have found high IGF-1 or IGF/IGFBP-3 ratio and high C-peptide to be associated with an increased risk of colorectal or colon cancer [12]–[19], [21]–[23]. Both insulin and IGF-1 may increase colorectal carcinogenesis through similar mechanisms and pathways, e.g. by increasing proliferation and decreasing apoptosis [3], [26]. In addition, insulin is known to regulate some aspects of the IGF-1 pathway, for example by decreasing IGF binding proteins and insulin and IGF-1 receptors form hybrids and there may be cross-reactivity for insulin and IGF at the receptor level [24], [25], [63], [64]. Interestingly, as we had observed previously in the NHS, in the combined NHS and HPFS cohort, individuals high in either insulin (C-peptide) or IGF-1/IGFBP-3 ratio appeared to be at higher risk but being high in both factors did not confer additional risk beyond being high in either one [21]. We speculate that this pattern suggests that either high insulin or high IGF-1 level is sufficient to activate the same pathway. This hypothesis merits further study and confirmation.

Insulin levels are readily modifiable by changes in diet and lifestyle e.g. by maintaining a healthy weight and increasing physical activity. Thus, in principle, healthy diet and lifestyle behaviors could potentially lower insulin levels thereby reducing risk of colon cancer [26]. On the other hand, for total IGF-1 levels modifiable dietary or lifestyle factors identified to date have been associated with differences of a relatively small magnitude [27]–[38]. In addition, in the HPFS we found that the strongest predictor of high IGF-1 are diets rich in major sources of animal protein, including low-fat milk, fish, and poultry, but not red meat, as well as total vegetable protein [37]. Many of these items are considered to be components of a healthy diet, so it remains unclear if dietary patterns that lower growth hormone and IGF-1 levels are desirable for adults for overall health. Thus, the finding that high 25(OH)D can substantially lower risk in individuals with high IGF-1/IGFBP-3 ratio or C-peptide levels is potentially important from a public health perspective.

We examined both multiplicative and additive interaction. Although most studies generally emphasize multiplicative interaction, additive interaction may be more relevant from a public health point of view because it better takes into account differences in baseline risk. For example, if a protective factor halves the risk of cancer in two groups, more cases can be prevented in the group with the highest baseline rate of cancer. We found that although higher levels of 25(OH)D might be desirable for all, the absolute number of preventable cases was greater in individuals with high IGF-1/IGFBP-3 molar ratio or high C-peptide levels. If high IGF-1 and high insulin underlie in part the high risk of colorectal cancer in Western countries, our findings suggest that vitamin D status may be one particularly important factor in such populations. Further studies are required to test this hypothesis.

The biologic factors underlying our observations are unclear. Some limited in vitro evidence suggests a direct link between the IGF-1/insulin axes and vitamin D. For example, laboratory studies on cultured the human prostate cancer cells the LNCaP human prostate cancer cell line have suggested that 1,25-dihydroxyvitamin D3 might influence IGFBP-3 levels [39], [40]. 1,25-dihydroxyvitamin D3 has been shown to increase expression of IGFBP-3 possibly by attaching to the BP-3-vitamin D response element (VDRE) on the IGFBP-3 promoter [40] suggesting that some of the cancer promoting effects of IGF-1 may be modified by vitamin D via changes in IGFBP-3 levels. Alternatively, IGF and insulin may be factors that accelerate colorectal cancer growth and inhibit apoptosis [3], [26], [65] and high 25(OH)D may be a factor that helps reduce proliferation and induce apoptosis through complementary pathways or mechanisms [66], [67].

In our study associations between IGF-1, the molar IGF-1/IGFBP-3 ratio and C-peptide and colorectal cancers were similar for men and women. However there is some evidence that positive associations between higher C-peptide/insulin levels and colorectal cancers may be more pronounced in men than in women [68]. Findings from a recent study also suggest that associations between components of the IGF axis and colorectal adenoma, a precursor for colorectal cancer [69], [70] may differ by sex [71]. In that study higher IGF-1 levels were positively associated with higher risk of colorectal adenoma in men while IGF-1 levels were not associated with colorectal adenoma in women.

Findings from a previous study from the NHS and HPFS suggest that either higher calcium intake or plasma 25(OH)D levels may be associated with lower fasting C-peptide levels, but after adjustment for BMI results were not statistically significant [72]. In that study, a non-significant (additive) interaction between calcium intake and plasma 25(OH)D levels with regard to C-peptide was also found, especially in men. We did not study interactions between calcium intake and components of the IGF and insulin axes with regard to risk of colorectal cancer because in our previous analyses on both cohorts [73] inverse associations between calcium intake and colorectal cancers were limited to distal colon cancers only and due to small numbers of distal colon cancers in this study statistical power was limited. Of note, in the large EPIC cohort with 1,121 colorectal cancer cases, moderate positive associations between IGF-1 levels and risk was observed only in those with low milk (and hence calcium) intakes [19]. This finding is consistent with a previous finding from the Physicians' Health Study where the positive association between IGF-1/IGFBP-3 molar ratio and colorectal cancer was strongest in men with low milk consumption [42]. Because calcium and vitamin D may interact [74], larger studies are required to examine these factors simultaneously in combination with IGF-1 and C-peptide levels.

One of the major strengths of this study includes its prospective design using blood samples that were obtained prior to diagnosis. In addition, due to our detailed follow-up and food frequency questionnaires we were able to take possible confounding due to known or suspected risk factors for colorectal cancer into account. One of the major disadvantages of this study is that blood samples were only obtained at one time point; however, in the HPFS, IGF-1, IGFBP-3 and molar IGF-1/IGFBP-3 levels were strongly to moderately correlated when measured in 149 samples donated about 3 years apart (Spearman partial correlation coefficient after adjustment for race: IGF-1 = 0.70, IGFBP-3 = 0.68, molar ratio = 0.59) [75]. For 25(OH)D the respective Pearson correlation coefficient after adjusting for age, race and season of the year was 0.70 (144 samples) [76]. Another limitation of our study was that because of the sample size and the examination of multiple factors, we had to stratify by medians to define “high” and “low” exposure for 25(OH)D, IGF/IGFBP3 molar ratio, and C-peptide, and we were unable to look at more extreme ranges (for example, high vs. low quintiles). Despite this limitation, we were able to observe statistically significant interactions on both the additive and multiplicative scales.

To our knowledge no epidemiological study has examined interactions between components of the IGF axis or C-peptide and plasma 25(OH)D levels with regard to risk of colorectal cancer. Therefore, the findings from this prospective study need to be confirmed in other studies before meaningful conclusions can be drawn. However, from a public health point of view, if confirmed these findings would provide a relatively easy way to reduce risk of colorectal cancer among those with high IGF-1/IGFBP-3 ratio or high C-peptide levels as vitamin D status is an easily modifiable risk factor.

In conclusion, if confirmed in other studies, the results from this study suggest that improving vitamin D status may help lower risk of colorectal cancer associated with higher IGF-1/IGFBP3 ratio or C-peptide levels.
